# Occupational influences on Spondylolysis and Spondylolisthesis in a cohort of 18-year-old male military conscripts

**DOI:** 10.1186/s12891-020-03747-8

**Published:** 2020-11-05

**Authors:** Oren Zack, Yair Barak, Aharon S. Finestone, Ayala Krakov, Dani Slodownik, Deborah Alperovitch-Najenson, Shlomo Moshe

**Affiliations:** 1 The Israel Defense Forces, Medical Corps, Tel-Aviv, Israel; 2 Department of Occupational and Environmental Medicine, School of Public Health, Sackler Faculty of Medicine, Tel-Aviv university, Tel-Aviv, Israel; 3 Department of Orthopedics, Shamir Medical Center, Zerifin, Affiliated to the Faculty of Medicine, Tel Aviv University, Tel-Aviv, Israel; 4 Department of Occupational Medicine, Hashfela and Jerusalem district, Maccabi Healthcare Services, 15 Rothschild St., Rishon Letzion, Israel; 5 Department of Dermatology, Tel Aviv Sourascky Medical Center, Tel Aviv, Israel; 6 Department of Physical Therapy, Recanati School for Community Health Professions, Faculty of Health Sciences, Ben-Gurion University of the Negev, Beer-Sheva, Israel

**Keywords:** Spondylolysis, Spondylolisthesis, Occupational, Low Back pain, Army recruits, Risk factor, Athlete, Adolescents

## Abstract

**Background:**

The reported prevalence of spondylolysis (SL) in the adult population is 6–7%. Data concerning adolescent-onset spondylolisthesis (SLS) and the impact of certain activities on it is scarce. We examined the risk of clinical progression of SL and SLS as a function of primary severity and occupational strain among military recruits.

**Methods:**

Based on the Israel defense Force (IDF) central human resources database, we identified 1521 18-year-old males inducted to the IDF with SL/SLS between the late nineteen nineties and early two-thousands. We followed changes in the SL/SLS status during the 3 years of obligatory military service. Disease severity was classified as Cat2: radiological findings of SL without clinical findings; Cat3: painful SL or asymptomatic grade 1 SLS; Cat4: grade 1 SLS with pain; Cat5: Grade 2 SLS. The soldiers were subdivided into the following occupational categories: administrative, combat, maintenance, and driving. The purpose was to compare the progression rates in different medical categories and job assignments.

**Results:**

There were 162 recruits in Cat2, 961 in Cat3, and 398 recruits in Cat4. The overall progression rate to Cat5 (grade 2 SLS) was 1.02%. Significant progression rates were seen amongst administrative soldiers with a relatively higher risk of progression from Cat4 (painful-grade-1 SLS: 2.2%) vs. Cat3 (asymptomatic SLS: 0.5%, relative risk = 4.7, *p* < 0.02). Other occupational categories did not exhibit significant progression rates.

**Conclusion:**

Progression of SL/SLS was highest in Cat4, i.e. for recruits already diagnosed with painful SLS (i.e. with a more severe baseline disorder). Progression did not correlate with military occupation. We recommend further follow-up studies that include, aside from progression rates, incidence rates of newly diagnosed grade 2 SL during military service.

## Background

The term spondylolisthesis (SLS) refers to slipping, or listhesis, of a vertebra (“spondylos” in Greek) relative to an adjacent vertebra. Spondylolysis (SL) refers to the dissolution of or a defect in the pars interarticularis of a vertebra [1]. The reported prevalence of SL in the adult population is 6–7% [2, 3]. In certain ethnic groups, the prevalence is considerably higher, which probably indicates a genetic predisposition [4, 5].

The natural progression of SL has been investigated in longitudinal studies through development to skeletal maturity. Fredrickson et al. followed 500 primary-school-aged children prospectively, demonstrating an incidence rate of 4.4% of SL at the age of six, progressing to 6% at skeletal maturity [2]; slip progression beyond skeletal maturity was rare in their cohort. A 20-year follow-up of adult patients with SL demonstrated that 40% of patients did not exhibit further slipping, 40% showed a slippage increase of 1-5 mm, and 5% of patients were observed as having significant further slippage [6].

Adolescent-onset SLS has been associated with various sports involving hyperextensions, such as gymnastics, cricket, and soccer and explained because of either an acute injury or a fatigue fracture resulting from repetitive stress exerted across the pars. Spondylolysis was more commonly seen in sports characterized by forceful and repetitive hyperextension-hyperflexion and rotation of the lumbar spine [7].

In contrast to the well-documented slippage progression in children and adolescents, adult progression of isthmic SLS has rarely been described or discussed in the literature. Overall, it was concluded that the risk of progression of SL, with or without low-grade SLS, to a more significant slippage, is rare [8]. The literature on this issue is deficient as no standard has been set to define what degree of slip progression is significant [8]. In occupational medicine literature, we found only one study published on the subject investigating the association between professional driving and SLS [9]. The study found that the rate of acquired SLS was higher among those driving > 15 years compared to those driving ≤5 years (7.1% compared to 1.1%, respectively) [9].

In the current study, we aimed to understand the contributing factors for developing grade 2 SLS, with pain and neurological findings in military conscripts. Conscripts were categorized as having radiological findings of SL without clinical findings or painful SLS grade 1. Different military job assignments represented varying occupational settings. We hypothesized the existence of a linear dose-response relationship between progression rates and higher-strain occupations.

## Methods

### Data source

The study cohort was a subgroup of all 17-year-old male Israeli military recruits reporting to the Israel Defense Forces (IDF) for pre-induction medical examinations between the end-nineties to the early two-thousands (the total numbers and exact time framework were not declassified). Data of the recruits’ pre-induction medical files were reviewed. All recruits answered a detailed medical questionnaire, followed by a face-to-face interview relating to their medical history and responses to the questionnaire. The recruits were asked directly about past or present musculoskeletal or back symptoms and morbidity. The physical examination was performed by a physician and included a thorough examination of the musculoskeletal system. Imaging and specialist referrals (usually an orthopedic surgeon) were utilized as necessary primarily for recruits with a previously unreported finding or with an unclear diagnosis. The referral included a detailed history and a physical examination supplemented by imaging and laboratory tests, as needed. The specialists were instructed to review any documentation and imaging and, as required, request further information, based on the date of assessment and how it coincided with the clinical presentation. As SL/SLS diagnoses require mandatory imaging, physicians could only encode the diagnosis if presented with a signed medical record and an imaging result. Any pars defect resulted in SL classification, so it was impossible to differentiate between unilateral and bilateral SL. Relatively recent radiographs were mandatory for higher grades of SLS. The follow-up period of 36 months aligned with Israel’s obligatory service period, comprised the occupational exposure period.

### Occupational categories

The soldiers were subdivided according to their military assignment into the following occupational categories**:** Combat Units (CU), Maintenance Units (MU), Driving Units (DU), and Administrative Units (AU). The CU service includes exceptionally strenuous physical activity and high mental stress. MU service is characterized by moderate physical activity and moderate mental stress (mechanics, welders, electricians and others), DU includes driving professions and includes either trucks or private vehicles. The AU service is predominantly characterized by sedentary office work, low mental, and no physical stress. We used these categories as markers for occupational stress.

### The medical Spondylolysis/spondylolisthesis categories

Recruits were classified at the recruiting offices into the medical SL/SLS categories (Cat1-Cat7) presented in Table 1, as previously described [10]. The grades of SLS refer to the measure of displacement whereby Grade 1: less than 25%; Grade 2: 25 to 50% and higher (referring to Grade 3: 50 to 75%; Grade 4: 75 to 100%; and Grade 5: complete). These categories were documented in the IDF central human resources database before induction and updated if any significant change in the soldier’s medical status occurred.

**Table 1:** Medical Spondylolysis/ Spondylolisthes categories assigned at recruiting offices, and updated throughout three years of army service. Each category was assigned based on the severity grading of Spondylolysis/Spondylolisthesis

Medical Categories	Cat1	Cat2	Cat3	Cat4	Cat5	Cat6	Cat7
Spondylolysis (SL)	No pathology	SL: an asymptomatic radiological finding	SL with pain				
Spondylolisthes (SLS)			Grade I asymptomatic SLS	SLS grade I with pain	SLS grade II or higher.	SLS with significant pain and activity limitations, but with a good prognosis.	SLS with significant neurological deficits and functional limitations.

Overall, the study population we followed for exacerbation included 1521 subjects who met the inclusion criteria (soldiers in SL/SLS category 1 were not included in our study). Soldiers in categories 2 to 3 were assigned to and served in all occupational categories. Soldiers in category 4 were generally restricted from CU and served in either MU, DU, or AU. Soldiers in category 5 served in DU or AU only. Those in category 6 and 7 were medically disqualified from mandatory service. Table 2 provides the corresponding percentages of assignments based on medical severity. The above assignment plan may have influenced our dose-response ratio due to the inverse relationship between disease severity and occupational strain – the highest severity soldiers (Cat.4) were denied the highest strain occupations (combat). Nonetheless, the fact that driving and maintenance professions, which are categorized by higher strain compared to administrative professions, and the similar percentage of soldiers assigned to these occupations serve to mitigate any significant bias in that aspect.

**Table 2:** Percentage of soldiers assigned to each occupational group by medical severity

Medical category	Administrative		Combat		Maintenance		Driving		Total	
	N	%	N	%	N	%	N	%	N	%
Cat2^a^	84	8.0%	64	23.3%	8	6.1%	6	9.5%	162	10.7%
Cat3	649	61.7%	208	75.6%	75	57.3%	29	46.0%	961	63.2%
Cat4^a^	319	30.3%	3	1.1%	48	36.6%	28	44.4%	398	26.2%
Total^b^	1052	69.2%	275	18.1%	131	8.6%	63	4.1%	1521	100%

Cat2 – Asymptomatic spondylolysis

Cat3 – Painful spondylolysis or Asymptomatic grade 1 spondylolisthesis without clinical findings

Cat4 – Grade 1 spondylolisthesis with pain

^a^Percentages in rows 2 to 4 are from total in assignment grouping

^b^Percentages in the bottom row are assignment grouping from the total cohort

### Follow up during the military service

All subjects were followed for 36 months by the units’ physicians noting any newly diagnosed conditions involving low back pain, SL or SLS. Any newly diagnosed illness or progression (based on complaint or injury) was documented by mandatory to referral to a military medical board that updated the SL/SLS category in the IDF central human resources database as was any change in duty assignment. The study endpoint per subject was defined as a change from the preliminary SL/SLS category (Cat2–4) to a higher category (Cat5 or beyond) during the three-year obligatory military service. This process was supervised and monitored by a trained physician stationed at the IDF medical corps headquarters.

### Data analysis

The relative risk (RR) calculated as the proportion of progression to Cat5 in Cat4 divided by the proportion of progression to Cat5 in Cat2 or Cat3, and Fisher’s exact 95% confidence intervals, are presented. The significance of point estimates was assessed using a χ^2^ test with correction for continuity. A *p* < 0.05 in two-tailed tests was considered to be significant. RR and 95% confidence intervals (CIs) were calculated using a standard statistical package (Compare2 version 1.28, Copyright JH Abramson 2000–2001).

## Results

The groupings of the cohort of 1521 conscripts identified with Cat2 to Cat4 SL/SLS are presented in Table 2. Progression to Cat5 during the 3 years of obligatory military service was seen in 16 subjects (1.02%). Table 3 delineates the various occupational groups’ contributions to this progression, primarily, the AU with its high proportion in the cohort (10/1052) and the combatants with the relative high progression rate (1.8%, 5/275, even though recruits with Cat4 were banned from CU). The RR for progression by job assignments are presented in Table 4. The RR for progression was higher in Cat4 than Cat3 only in AU (RR = 4.7, *p* < 0.05). The higher difference between occupational groups was found between CU and MU (RR = 1.8/0.8 = 2.4, CI = 0.3–20.2, NS), but without statistical significance. There was no progression amongst the drivers, but this is not significant as there were only 63 in DU.

**Table 3:** Progression of spondylolysis or grade-1- spondylolisthesis (Categories 2–4) to grade-2- spondylolisthesis or higher (Cat5-Cat7) by job assignments

Categories	Administrative		Combat		Maintenance		Driving		Total	
Cat2	0/84	0%	2/64	3.1%	0/8	0%	0/6	0%	2/162	1.23%
Cat3	3/649	0.5%	3/208	1.4%	1/75	1.3%	0/29	0%	7/961	0.73%
Cat4	7/319	2.2%	0/3	0%	0/48	0%	0/28	0%	7/398	1.76%
Total	10/1052	1.0%	5/275	1.8%	1/131	0.8%	0/63c	0%	16/1521	1.02%

Cat2 – Asymptomatic spondylolysis

Cat3 – Painful spondylolysis or asymptomatic grade 1 SLS without clinical findings

Cat4 – Grade 1 Spondylolisthes with pain

**Table 4:** Relative risk for progression between categories in different job assignments*

Category comparison	Administrative	Combat	Maintenance	AU + CU + MU combined
Category 2➔5	NR**	NR	NR	0.59 (0.11 to 5.84)
Category 3➔5	NR	0.46 (0.1–2.7)	NR	1.42 (0.27 to 14.27)
**Category 4➔5**	4.7 (1.2–18.2) ***	NR	NR	2.41 (0.72 to 8.20)
All Categories (2 to 4)➔5	1.0%	1.8%	0.8%	1.02%

*Columns describe job allocations except for driving where there were too few subjects and no progression

Rows are according to baseline SL/SLS category, with the bottom line summing all categories

** NR is Non-Relevant, indicating too few subjects for relevance

*** *p* < 0.05

## Discussion

In this study, we investigated the progression rates to SLS grade 2 from SL and SLS grade 1 in a population of 1521 recruits according to their profession (CU, MU, DU, AU) during 36 months follow up. The main findings were that the incidence rate of progression of SL or SLS grade 1 to SLS grade 2 was 1.02% and that the RR for developing severe SLS was higher in category 4 compared to category 3 in AU (RR = 4.7, *p* < 0.05). The overall incidence of SL/SLS in the population from which the cohort was drawn can not be published, though the SL/SLS categories’ overall incidence may be seen in Bar Dayan et al. publication [10].

In skeletally immature individuals, the tendency for lumbosacral slip progression is well known. Slip progression is most likely to occur in adolescents younger than 15 years of age, usually during the adolescent growth spurt [11]. In contrast to the well-documented slip progression in children and adolescents [3, 7, 8], especially in athletes [7], the adult progression of isthmic SLS has rarely been described or discussed in the literature [11, 12]. Some authors even dispute its existence and clinical importance [2, 8]. Harris and Weinstein [12] reported the outcome of 11 patients in whom Grade-III and IV SLS were treated non-operatively over an average eighteen-year follow-up and found that 5/11 had one or more neurological findings, but none were incontinent. In his work, Floman [11] described 18 patients, ages 32 to 55 years, with documented adult isthmic slip progression who reported incapacitating low back pain, accompanied in most by significant sciatica. Documented slip progression ranged from 9 to 30% (average, 14.6%), and occurred from 2 to 20 years. Seitsalo et al. [13] followed-up 272 patients with SLS aged 14.3 for 14.9 years and found that 23% of adult patients exhibited some slip progression. Virta and Osterman [14] reported a 5.6% slip increase over a 17-year follow-up in 40 adult patients with SLS who had not undergone operations. Our study reflects a mean annual progression of 0.34% compared to 2% [6], 1.5% [12] and 0.32% in other studies. The variance between incidence rates emerges from different severity classifications, different age groups, and different follow-up times. Fredrickson et al. [2] estimated that approximately 15% of individuals with a pars interarticularis lesion had progression to SLS. The slip was seen predominately during the growth spurt, with minimal change after sixteen years. Others have accepted this concept [8]. Our cohort of 1521 patients is larger than all the previous studies [11–14] summed together (341 patients) and proves that the risk of progression of SL or low-grade SLS to a more significant slip is not ignorable and is 1.02% in 3 years among 18-year-old males.

We had hypothesized that there would be different progression rates in different severity scales of SL/SLS patients, specifically that the progression rate would be higher in Cat4. The low progression rate enabled statistical significance only in the large sample group (AU, that comprised two-thirds of our study population). In this group, the incidence of progression rate to Cat5 (SLS Grade 2 or higher) from Cat3 (Painful SL or asymptomatic grade 1 SLS) and category 4 (Grade 1 SLS with pain) was 0.5 and 2.2%, i.e. the RR was 4.7 (*p* < 0.05). This significance did not appear in the overall calculations, possibly due to the exclusion of Cat4 in CU. We did not find similar results in other studies; however, the incidence in Seitsalo et al. [13] for SLS patients’ progression was 2% yearly but without any comparison within subgroups.

We also hypothesized to find higher CU progression rates than AU and MU since they have more physical and strenuous activities and training during their services. It is known, that sports professionals have a significantly greater prevalence of SL and SLS, for instance, gymnastics (30%), American football (20%), weight lifting (23–30%), and wrestling (30–35%) [7, 11, 15]. The literature also shows an approximately fourfold increase of SL and SLS in adolescent dancers than the general population [16]. This increased incidence is mainly attributed to the repetitive shear forces of hyperextension positions and the poor mechanics and alignment often used by young dancers to obtain their positions. Ishimoto et al. [17] found, in a nested case-control study (722 vs. 605, mean age 70), that occupational driving and working in the agricultural/fishing industry were associated with radiographic spondylolisthesis (Odds ratio: 2.4 and 3.5, respectively). We indeed found a difference in the incidence rate between CU (1.8%) to AU (1%) and MU (0.8%), but those differences were not statistically significant. We estimate that the difference in activities and the short period of follow-up is the reason for the negative results.

At first glimpse, it not clear why the only subgroup that progressed with a significant RR was the AU. Two factors may contribute to this: almost 70% of the cohort was in AU; the second largest group was CU, from which Cat4 was banned.

The present study has several strengths. The first being that it is based on a large cohort (1521 subjects). Second, SL and SLS’s definition is exact and decided by a military medical committee based to a great extent on imaging (particularly crucial for Cat5 - SLS grade 2, since it is dependent on imaging). Third, SL/SLS data were collected from computerized data, guaranteeing a high coverage of progression. Fourth, all soldiers were conscripts in obligatory service of uniform age, rank, living conditions, and diet provided for each occupation.

There are several limitations to this study. We could not publish the actual number of subjects from which our cohort was drawn due to military censor restrictions. The study includes only male recruits, and therefore the conclusions are limited to males and possibly soldiers in general. The tendency to allocate higher grades of SL/SLS to less strainful occupations could have caused bias. Since the incidence of progression from SL to SLS is generally small, the size of our cohort, despite being much more prominent than other studies, is still not big enough to provide overall statistically significant results, beyond the stated subgroups. Moreover, despite collecting the baseline data prospectively, our study is retrospective. Data was not collected for research purposes and prevented differentiation between unilateral and bilateral SL and might have skipped asymptomatic SL/SLS progression.

## Conclusions

We found that progression from SL or low-grade SLS to a painful or high-grade SLS during early adulthood (ages 18–21) is 0.34% yearly. We found some clues which support the hypothesis that the higher the severity scale, the higher the RR. It seems that the type of duty has an effect, but since the follow-up was too short, and the activities were not equivalent to those of elite sportspeople, the results were not conclusive. Our data do not support changing the IDF human resources criteria for job allocation. More research is needed, with longer follow-up and larger groups, to understand SL and SLS’s occupational implications in different jobs better.

## Data Availability

The data is kept at the IDF, Medical Corps. The raw data cannot be shared due to limitations on exporting data form military databases.

## Abbreviations

IDF: Israel Defense Forces
CU: Combat Units
MU: Maintenance Units
AU: Administrative Units
SL: Spondylolysis
SLS: Spondylolisthesis
RR: Relative Risk
CI: Confidence interval
